# Organ Involvement in COVID-19: A Molecular Investigation of Autopsied Patients

**DOI:** 10.3390/microorganisms10071333

**Published:** 2022-07-01

**Authors:** Prem Shankar, Jitendra Singh, Ankur Joshi, Anvita Gupta Malhotra, Arti Shrivas, Garima Goel, Priyal Gupta, Jayanthi Yadav, Saurabh Saigal, Sarman Singh, Shashank Purwar

**Affiliations:** 1 Department of Microbiology, All India Institute of Medical Sciences, Bhopal 462020, India; premshankar.1506@gmail.com (P.S.); anvitagupta16@gmail.com (A.G.M.); arti2414@gmail.com (A.S.); priyalgupta818@gmail.com (P.G.); sarman.singh@gmail.com (S.S.); 2 Department of Translational Medicine, All India Institute of Medical Sciences, Bhopal 462020, India; jattinsingh@gmail.com; 3 Department of Community and Family Medicine, All India Institute of Medical Sciences, Bhopal 462020, India; ankur.cfm@aiimsbhopal.edu.in; 4 Department of Pathology & Laboratory Medicine, All India Institute of Medical Sciences, Bhopal 462020, India; garima.patho@aiimsbhopal.edu.in; 5 Department of Forensic Medicine, All India Institute of Medical Sciences, Bhopal 462020, India; jayanthi.fmt@aiimsbhopal.edu.in; 6 Anaesthesia and Critical Care, All India Institute of Medical Sciences, Bhopal 462020, India; saurabh.criticalcare@aiimsbhopal.edu.in; 7 Department of Biomedical Sciences, Indian Institute of Science Education and Research (IISER), Bhopal 462066, India

**Keywords:** SARS-CoV-2, autopsy, qRT-PCR, organ involvement

## Abstract

Precise reasons for severe manifestation of SARS-CoV-2 remain unanswered, and efforts have been focused on respiratory system management. Demonstration of unequivocal presence of SARS-CoV-2 in vital body organs by cadaver autopsy was the only way to prove multi-organ involvement. Hence, the primary objective of the study was to determine presence of the SARS-CoV-2 in various organs of patients succumbing to SARS-CoV-2 infection. A total of 246 samples from different organs of 21 patients who died due to severe COVID-19 illness were investigated by qRT-PCR, and SARS-CoV-2 was detected in 181 (73.57%) samples and highest positivity of SARS-CoV-2 being (expectedly) found in nasopharynx (90.4%) followed by bilateral lungs (87.30%), peritoneal fluid (80%), pancreas (72.72%), bilateral kidneys (68.42%), liver (65%) and even in brain (47.2%). The deceased patients were categorized to three subgroups based upon the extent of organs in which SARS-CoV-2 was detected by qRT-PCR (high intensity ≥80%, intermediate intensity = 65–80% and low intensity ≤65% organs involvement). It was conclusively established that SARS-CoV-2 has the property of invasion beyond lungs and even crosses the blood–brain barrier, resulting in multi-system disease; this is probably the reason behind cytokine storm, though it is not clear whether organ damage is due to direct injury caused by the virus or result of inflammatory assault. Significant inverse correlation was found between the Ct value of lung samples and number of organs involved, implying that higher viral load in lungs is directly proportionate to involvement of extrapulmonary organs and patients with higher viral load in respiratory secretions should be monitored more closely for any warning signs and the treatment strategies should also address involvement of other organs for better outcome, because lungs, though the primary site of infection, are not the only organ system responsible for pathogenesis of systemic illness.

## 1. Introduction

SARS-CoV-2 is a single-stranded enveloped RNA virus responsible for causing the current pandemic of respiratory illness [1,2]. COVID-19 was first reported at Wuhan, China, and spread rapidly to many countries across all six WHO regions. As of 25 March 2022, the pandemic has resulted in 476,374,234 cases and 6,108,976 deaths worldwide [3]. The first case from India was reported in Kerela, Thrissur on 27 January 2020 [4]. The most common symptoms associated with COVID-19 illness are fever, cough, expectoration, headache, and myalgia or fatigue; however, gastrointestinal involvement and dyspnea are associated with severity at the time of hospital admission [5]. A high probability of transmission of the virus is also reported in first 3 days (incubation time, which can extend to 24 days in some patients) of infection even though the patient is asymptomatic [6]. Acute infection is primarily diagnosed by detecting viral ribonucleic acid (RNA) in respiratory secretions collected from clinical cases [7] and it has been reported that in case of deceased patients also, the presence of viral RNA can be detected by quantitative real-time reverse transcription–polymerase chain reaction (qRT-PCR), considered as the gold standard for diagnosing COVID-19 infections [8,9].

A qRT-PCR assay positive for SARS-CoV-2 is considered one of the key decisive factors for isolation/hospitalization of symptomatic patients and for quarantining immediate contacts [10], even though qRT-PCR assay cannot distinguish between infectious and non-infectious virus particles.

The cycle threshold (Ct) value of qRT-PCR is the number of amplification cycles required for the fluorescence signal to exceed the basal threshold level. It is a relative measure of the target concentration in the PCR reaction. Attempts have been made to quantify the viral load that is based on Ct values in the qRT-PCR for COVID-19 diagnosis and, thereby, virus replication and transmission kinetics [11,12], despite acknowledgment of associated variables and the published studies illustrating the correlation between the Ct values and virus cultivability [13]. However, the precise pathophysiological mechanisms behind the morbidity and mortality associated with involvement of vital body organs in reference to viral load resulting in systemic COVID-19 illness is yet to unfold and not very well understood as to how an airborne pathogen that has a strong affinity for the respiratory system elicits an overwhelming systemic inflammatory response.

Post-mortem study is an indispensable way to learn about the pathobiology of the disease and is an immensely valuable tool for documenting the presence of a pathogen in various organs and thus providing insight to the involvement of multiple organs and genesis of the immune response. This study was conceived to elucidate whether SARS-CoV-2 has ability to invade various organs after primary lodgment in upper respiratory tract through droplet infection and which organs get involved. This valuable information will add to the increasing data in this area of pathophysiology and subsequently developing treatment modalities [14,15,16,17,18,19,20,21,22,23,24,25]. As of now, no confirmed case of COVID-19 infection from the dead body has been reported [26]; however, it will be premature to comment on whether SARS-CoV-2 transmission happens from the infected dead body to the laboratory, hospital, mortuary workers and to close family relatives though there has been fear among these groups of people about the transmission. Even local government agencies, in order to prevent any potential transmission, had issued guidelines for carrying dead bodies from the hospital to cremation facilities directly without performing customary social last rites, which also include bathing of body in various communities. Therefore, international guidelines emphasize the use of Biosafety level (BSL) containment for cadavers’ investigation in case of suspected or confirmed COVID-19 infection [27]. Therefore, the aim of the present study is to unravel the SARS-CoV-2 systemic dissemination and pathogenicity by means of molecular diagnosis in a major organ system of patients who died due to COVID-19 infection.

## 2. Materials and Methods

### 2.1. The Study Cases

All 21 autopsy cases enrolled in this study were deceased patients that were critically ill with COVID-19, the diagnosis for which was made by detecting the presence of SARS-CoV-2 in upper respiratory secretions by RT-PCR test in DHR-ICMR Regional Virology Laboratory in the department of Microbiology at the All India Institute of Medical Sciences, Bhopal, Madhya Pradesh (India). The consent for performing autopsy was obtained from the relatives of the deceased after explaining the utility of the study in the realms of science and, while we approached relatives of 134 deceased patients, consent was received from 21 cases, which is understandable given the situation of pandemic-stricken grieving families.

### 2.2. Study Settings and Ethical Consideration

This prospective study was conducted from August 2020 to October 2020 in the Department of Microbiology, and Forensic Medicine & Toxicology, AIIMS-Bhopal, with prior ethical clearance from Institutional Human Ethics Committee (IHEC) of AIIMS-Bhopal (India) (Approval No.: IHEC-LOP/2020/IM0273).

### 2.3. Autopsy and Collection of Samples

A total of 21 cases who died of COVID-19 infection were included in this study and acute respiratory distress syndrome (ARDS) score at the time of admission was obtained. One patient was declared brought dead to the hospital, therefore for this patient ARDS status at the time of admission was not available. The routine autopsy of COVID-19-infected cases was not permitted by the Indian Council of Medical Research (ICMR), therefore these autopsies were performed only for research pursuit after obtaining approval from the Institute Ethical committee of AIIMS—Bhopal.

Since no specific guidelines were available for COVID-19 autopsies, the procedures were performed as per guidelines for highly infectious autopsy cases given by the Center for Disease Control and Prevention (CDC) [28], Royal College of Physicians (RCP) [29], COVID-19: Guidelines on Dead Body Management [30], and World Health Organization (WHO) [31,32]. Since these autopsies were performed on the cadavers infected with SARS-CoV-2, which is potentially infectious and highly transmissible, movement in and out of the autopsy room was strictly limited. Availability of all the prerequisites for the procedure (instruments and reagents) was ensured inside the autopsy room before commencing the autopsy. These include scalpel, trays, knives, syringes, forceps, camera, 10% neutral buffered formalin, normal saline, 1% sodium hypochlorite, rectified spirit and data entry forms. The Viral Transport Medium (VTM) vials (HIMEDIA, HiViral^TM^ Transport Medium, AL167, Maharashtra, India) for transfer of autopsied samples to the Microbiology Laboratory for detection of SARS-CoV-2 by qRT-PCR were labelled. The autopsies were performed in an isolated autopsy room equipped with HVAC, having 12 air cycle changes per hour (ACH) and high-efficiency particulate air (HEPA) filters. Separate zones (clean, buffer, and dirty) were maintained inside the autopsy room to limit the spread of infection. Low aerosol-generating techniques were employed during the autopsy, which included the usage of handheld instruments (chisels, hammers, and rib shears) instead of an electric saw, and the areas being dissected were covered by gauze to minimize spillage and splashes. Care was taken to open only one cavity at a time; 1% sodium hypochlorite solution was sprayed on the body at each step after collection of samples and the knife and forceps were sterilized with the spirit or a new knife was used before each sampling to prevent carryover contamination.

In total, 246 organ samples, which includes nasopharynx, bilateral lungs, pancreas, bilateral kidneys, liver, spleen, brain, uterus, peritoneal fluid, and pericardial fluid, from 21 dead bodies were collected in the VTM vials. The samples were then packed and transported to the Department of Microbiology, AIIMS—Bhopal and stored at −80 °C until further processing. Autopsied samples from lungs, liver and kidney tissues were also subjected to determine histopathological changes, if any. The interested readers can refer [33] for clinical and histopathological findings.

### 2.4. Processing of the Samples for SARS-CoV-2 RNA Extraction

Prior to the processing, the samples stored at −80 °C were thawed to room temperature. Samples were segregated into two categories, namely body fluids/swabs and tissue samples, and processed in Biosafety cabinet type 2 level B2. RNA extraction was done by using the RNeasy Mini kit, Part 1 (Qiagen #Cat. No. 74104, Hilden, Germany).

Each autopsied tissue was cut into small pieces aseptically and approximately 30 mg of tissue was taken and homogenized in sterile mortar and pestle by using 600 µL of buffer RLT, which was provided by the kit manufacturer. The samples (body fluids/swabs) in the VTM vials were also aliquoted in microcentrifuge tubes and 600 µL of buffer RLT was added into the suspension. Lysates were processed further as per protocol provided by the kit manufacturers. All the extracted RNA were quantified by using Qubit™ RNA HS Assay Kit (ThermoFischer’s#Cat. No.: Q32852, Waltham, MA, USA) prior to SARS-CoV-2 detection.

### 2.5. SARS-CoV-2 qRT-PCR

The qRT-PCR was performed by using extracted viral nucleic acid. Detection of SARS-CoV-2 RNA was done in a 30 µL reaction volume by using real-time fluorescent RT-PCR kit (BGI genomics #Cat. No. MFG030010; Copenhagen, Denmark, Europe) as per the manufacturer’s protocol using real-time PCR System (ABI-7500, Applied Biosystem, Waltham, MA, USA). The β-Actin (a human housekeeping gene) was used as internal control. Briefly, the qRT-PCR reactions were carried out by setting up the program as per manufacturer’s protocol which briefly include 50 °C for 20 min for the reverse transcription, followed by incubation at 95 °C for 10 min, and 95 °C for 15 s to 60 °C for 30 s for 40 cycles. The results of the test were interpreted as per baseline and threshold value of the reference gene as provided in the kit by the manufacturer.

### 2.6. Ct Value and Viral Load in Target Body Organs

The positive and negative controls available in the kits were run along with the test samples. The samples having Ct value < 38 were considered positive, as per the manufacturer’s protocol (determined using ROC of standards and tested samples). The target for the FAM channel was ORF1ab gene and in the HEX channel was the human gene (IC) RPP30. While, the specimens were considered negative, if the Ct of FAM was zero or ‘Not Available’ with Ct at VIC/HEX not higher than 32 and also, if the standard curve for FAM was not Sigmoidal shaped. All tests were run in a set of duplicates [Supplementary Material].

### 2.7. Categorization of Cases and Corresponding Organ Samples

All the recruited cases were classified into three groups on the basis of the number/proportion of the organ samples involvement: High, Intermediate or Low. ARDS category at the time of admission was taken into consideration as per Berlin definition, which is defined by: timing (within 1 week of clinical insult or onset of respiratory symptoms); radiographic changes (bilateral opacities not fully explained by effusions, consolidation, or atelectasis); origin of edema (not fully explained by cardiac failure or fluid overload); and severity based on the PaO2/FiO2 ratio on 5 cm of continuous positive airway pressure (CPAP). The 3 categories are mild (PaO2/FiO2 200–300), moderate (PaO2/FiO2 100–200), and severe (PaO2/FiO2 ≤ 100) [34]. This data for organ involvement and ARDS Category at the time of admission was correlated with their RT-PCR Ct values, and number of days.

### 2.8. Statistical Analysis

The data containing the clinical details and the organ-wise Ct value for the cases was studied and analyzed in R [35]. All the cases were categorized into 3 subgroups based on organ involvement in SARS-CoV-2 infection. The Friedman test was applied to understand whether there are any statistically significant differences in Ct values amongst the distributions of these three subgroups classified as high, intermediate, and low intensity on the basis of number of organs involved after accounting for the type of organ involved. For the purpose of analysis, only 8 organs sies (Brain, Trachea, Kidney, Liver, Lung, Nasopharynx, Pancreas and Peritoneal fluid) were considered because these were the organs harvested from all 21 autopsied cases. Further, the ggplot2 function was used to generate the boxplot to represent the organ wise distribution of data in three subgroups based on ORF1ab gene Ct value. Since the data distribution was non-parametric, Spearman Rank Correlation test was applied to determine the relation between the lung tissue Ct value and percentage of organ involvement in each case.

## 3. Results

The clinical autopsies were conducted at the All India Institute of Medical Science—Bhopal, which is a tertiary care central government institute designated as Institute of National Importance (INI) and located in state of Madhya Pradesh (Central India), which was amongst the worst-affected states by COVID-19 pandemic. This study was done primarily to understand the virulence of SARS-CoV-2 in terms of ability to invade various organs beyond upper respiratory tract in COVID-19 patients and also to determine any correlation between organ involvement, ARDS category at the time of admission to the hospital, and survival days of the autopsied study cases.

### 3.1. Patient, Sample Type and qRT-PCR Positivity in Recruited Cases

A total of 21 cases were recruited in the study. This includes 15 male and 6 female cases who died with SARS-CoV-2. Maximum duration of survival was 39 days for these 21 cases and ARDS category as per Berlin definition was determined at the time of admission, except for one patient who was declared brought dead on arrival to the hospital facility. The age of the study cases ranged from 25–84 years. A comprehensive autopsy was performed on 21 cases for determining presence of SARS-CoV-2 in various organs by qRT-PCR.

**Figure 1:**
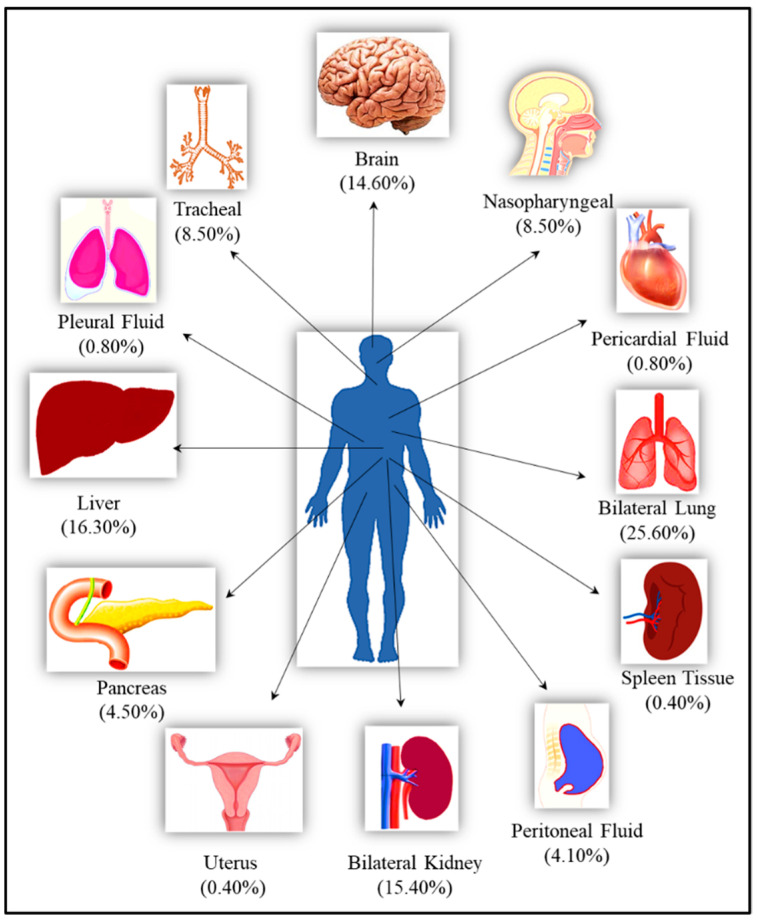
The list of the organs from the autopsied bodies along with their percentage representation in the total studied samples.

In total, 246 samples, which included samples from nasopharynx, bilateral lungs, trachea, peritoneum, pancreas, bilateral kidney, liver, brain, pericardium, pleura, spleen, and uterus, were collected from 21 cases and studied. For some organs such as brain, lungs and liver, samples were collected from multiple sites from the same case. The qRT-PCR assay was positive for 181 (73.57%) samples while 65 (26.42%) were negative Figure 1.

**Figure 2:**
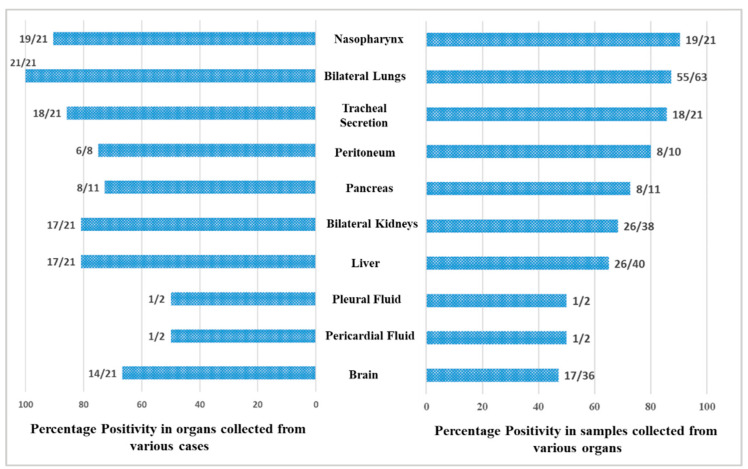
Positivity of the organ samples from the autopsied patients.

A total of 246 samples from various vital organs were collected during autopsy and their RT-PCR results shows that the SARS-CoV-2 was detected from all organs. The percentage positivity diagnosed by RT-PCR of autopsied specimen collected from 21 cases is illustrated in Figure 2. The bar chart on the right represents percentage positivity of the organs and on the left depict the number of cases in which the organs were positive for the present of SARS-CoV-2 RNA. For SARS-CoV-2 infection, as it is primarily responsible for causing respiratory illness, the highest detection was expectedly observed in nasopharyngeal samples (90.5%) followed by lungs (87.3%) and trachea (85.7%). SARS-CoV-2 was detected in peritoneal (80%), pancreas (72.7%), kidney (68.4%), and liver (65%) samples, and also could pass the blood–brain barrier, seen in nearly 50% of the samples. Of the total 21 cases autopsied and studied, the presence of SARS-CoV-2 was seen in lungs for all the cases, liver for 17 cases, in pancreas for 8 cases, in peritoneal fluid for 6 cases, and in brain for 14 cases.

### 3.2. Categorization of Study Sample Based on Organ Involvement

**Figure 3:**
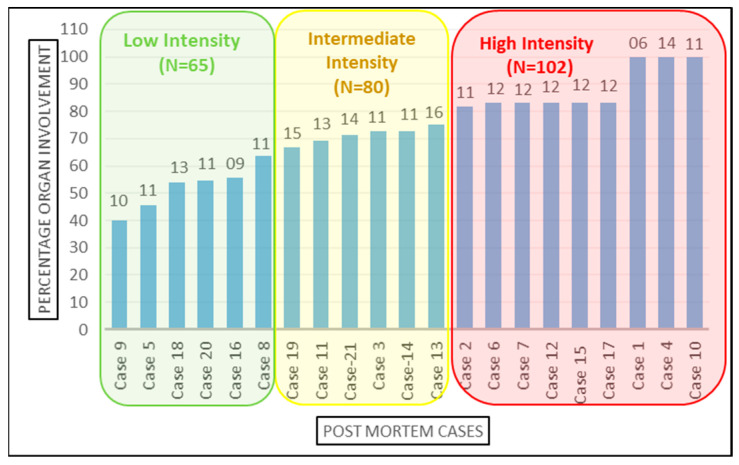
Categorization of the cases based on organ involvement. Numbers on top of the bars represents the total number of samples studied from the corresponding case.

The 21 cases were further classified into high-, medium-, and low-intensity infection based on the number of organs involved Figure 3. Organ involvement was determined as the percentage of organs tested positive for SARS-CoV-2 infection by RT-PCR out of total number of samples collected from that autopsied case. As per equation below, the organ involvement for all 12 organs studied was calculated for each case. The minimum and maximum percentage organ involvement of autopsied case for SARS-CoV-2 were 40% and 100%, respectively. For internal comparison, cases with <65%, 65–80% and >80% positivity were designated as ‘low’-, ‘intermediate’- and ‘high’-intensity organ involvement, respectively. There is no precedence available in published scientific literature for classifying SARS-CoV-2 patients’ in high-, intermediate- and low-intensity organ involvement based on the number of organs involved, because the insights to unprecedented SARS-CoV-2 infection are still evolving and our study is amongst very few, and is the only study to emerge from Indian sub-continent, which aimed to demonstrate the dissemination of SARS-CoV-2 from lungs to other vital organs. Therefore, this can serve as a reasonable reference analysis tool in future autopsy studies.


$$\text{Organ Involvement}=\sum_{n=1}^{n=12}(\frac{\text{Organ samples tested positive for SARS-CoV-2}}{\text{Total Number of samples tested for that organ}})100$$

**Figure 4:**
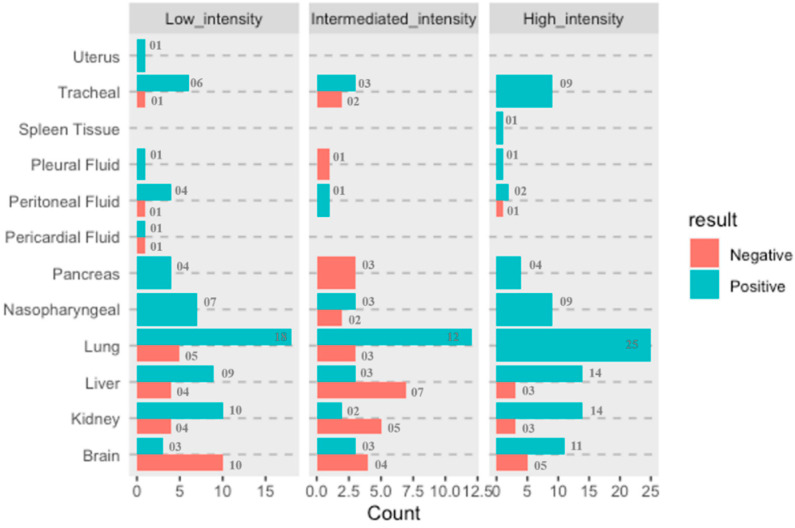
Representation of the proportionate of organ involvement and COVID-19 viral load intensity (low, intermediate, and high).

RT-PCR Ct value is used as a surrogate marker for the viral load in the samples and therefore correlated with the infectivity of the patient. However, studies have shown that Ct value independently cannot determine the SARS-CoV-2 virus load since the virus has been successfully isolated from the samples with Ct value > 32 [36], implying that the Ct value cannot be an independent predictor of virus load and transmissibility because more than one of the variables are associated in Ct load calculation and multiple factors are integral to transmission dynamics. However, to understand whether there are any statistically significant differences of Ct values amongst the distributions of these three groups, namely high-, intermediate- and low-intensity cases, Friedman test was applied. For analysis purpose, we had taken only those eight organ sites (brain, bilateral kidneys, liver, bilateral lungs, nasopharyngeal, pancreas, peritoneum, tracheal samples) that were extracted and tested from all 21 cases Figure 4.

It was observed that proportionate involvement of all organs considered in this study/detection of SARS-CoV-2 virus in various organs is directly proportional to the infection intensity except for the brain, from which SARS-CoV-2 was detected in a greater number of patients classified as having low-intensity infection compared to patients identified as having intermediate intensity. This study is probably the first to demonstrate the ability of SARS-CoV-2 to reach the brain by crossing relatively impermeable blood–brain barrier, and further investigations will be required to establish the involvement of the brain w.r.t. in severity of infection caused by SARS-CoV-2.

**Figure 5:**
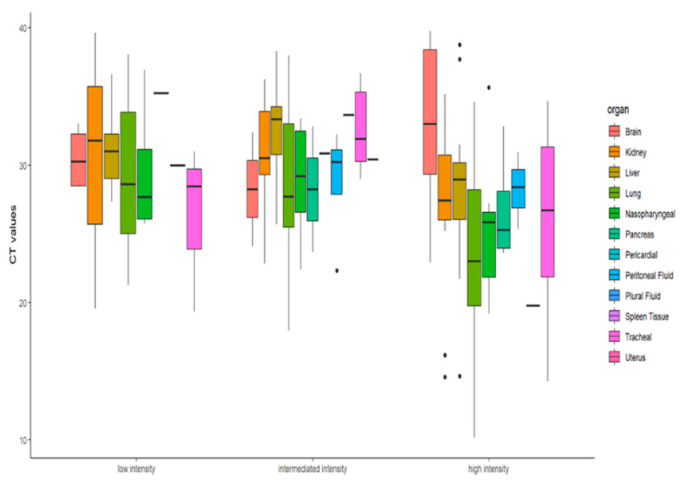
Boxplot representation of the Ct values of infected organs as per the organ involvement.

Descriptive statistics were performed to find whether the RT-PCR Ct value can determine the infection intensity of COVID-19 patients. The Ct values differences were not found to be significant (x^2^ = 4.75, df = 2, *p* = 0.0930) at 95% CI. As reported in various studies, the Ct value depends on variable factors and is not an adequate marker for the viral load and infectivity. Similarly, our findings too corroborate that the extent of infection in terms of organ involvement cannot be accessed solely on the basis of Ct value. The molecular findings in terms of presence of SARS-CoV-2 viral RNA in autopsy samples did not show any significant correlation with organ involvement of the study cohort. Figure 5’s boxplot also shows that for low- and high-intensity infection the Ct value difference is not significant.

**Figure 6:**
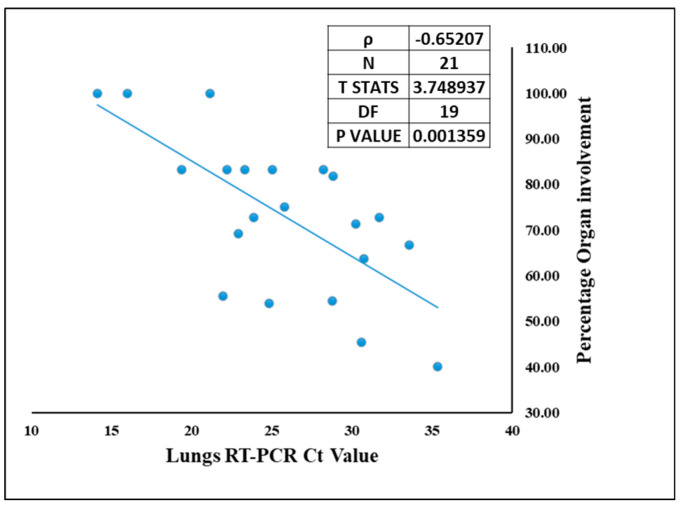
The correlation between SARS-CoV-2 viral RNA Ct value in the lungs and the organ involvement in each case.

Though COVID-19 is associated with the respiratory system, we demonstrate presence of SARS-CoV-2 other vital organs as well. The site of lodgment for SARS-CoV-2 is nasopharynx, which being contiguous to lungs makes them the primary target organ, and lungs were the only organ from which SARS-CoV-2 was detected by RT-PCR in all 21 autopsied cases. Hence, correlation could be drawn only between the Ct value of SARS-CoV-2 in lungs and organ involvement. The organotropism associated with this infection is a direct function of the viral load in the lungs. This means that higher the viral load in the lungs (lower Ct value), the more the infection will spread in other extrapulmonary organs [37]. Therefore, the percentage of organ involvement for each case was correlated with viral RNA in the lungs (lungs qRT-PCR Ct value) Figure 6. Spearman’s rank correlation test showed a negative correlation of −0.65 with T statistics of 3.74 and significant *p* value of 0.0013. This suggests that higher pulmonary viral RNA (i.e., lower Ct value) corresponds to high percentage of organ involvement, contributing to the broad virus dissemination in case of SARS-CoV-2.

### 3.3. ARDS Categories and Intensity of Organs Involved

**Figure 7:**
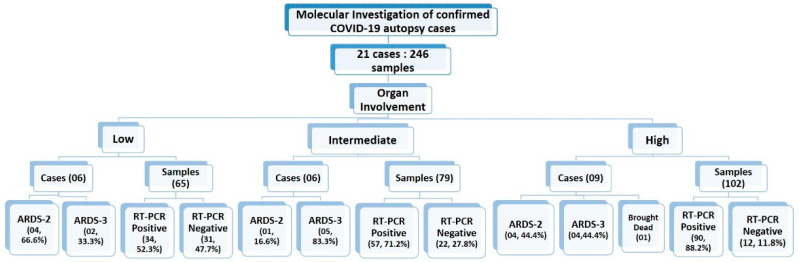
Summarizing the study cases and samples classification based on organ involvement, RT-PCR status and ARDS categories.

For all the recruited cases, the data for ARDS category was assigned at the time of admission. The detailed distribution of the study cases based on organ involvement along with their ARDS categorization and qRT-PCR results is illustrated in Figure 7. Duration of the survival and ARDS category were also documented for all the 21 cases. Among all these cases, only one was declared brought dead (Case No.10) to the hospital, whereas the remaining 20 cases were admitted to the COVID-19 Intensive Care Units of AIIMS—Bhopal, Madhya Pradesh (India). Mean survival days for the study cases was 9.2 days. The highest hospital occupancy was seen for Case 5, which was 39 days, and the case belonged to the subgroup of low-intensity organ involvement. Lowest survival was seen as 2 days in two cases that were in intermediate- and high-intensity organ involvement, which could indicate that the short duration during which these patients succumbed to death was due to higher organ involvement. There was no significant correlation observed between the ratio of organ involvement and the days; however, the mean days for cases with low organ involvement was higher (14.41) as compared to the cases with high organ involvement (7.18), which is reflective of the severity associated with the organ involvement. Fundamentally, it is not only the function of disease severity proxied by the ratio of organ involvement alone, but also the baseline demographic characteristics, co-morbidity status, access to care, decisions protocols, background vulnerability, and, above all, idiosyncratic factors that are beyond the scope of this study. However, this study was conducted in the early days (1st wave) of the SARS-CoV-2 pandemic and on a small subset of 21 cases deceased due to SARS-CoV-2. Moreover, there exists a probability that Case 5, which survived for 39 days, has influenced the conclusion drawn on mean days. Therefore, similar and subsequent studies may further add to this domain of survivability after SARS-CoV-2 infection, which will still be influenced by disease endemicity leading to the presence of antibodies either due to natural infections or advancements in prophylactics and also influenced by the availability of more effective therapeutic agents. Therefore, studies conducted after stabilization of epidemiological determinants will be able to conclude the correlation between the organ involvement and days.

### 3.4. Different Body Organs as per ARDS Category

**Figure 8:**
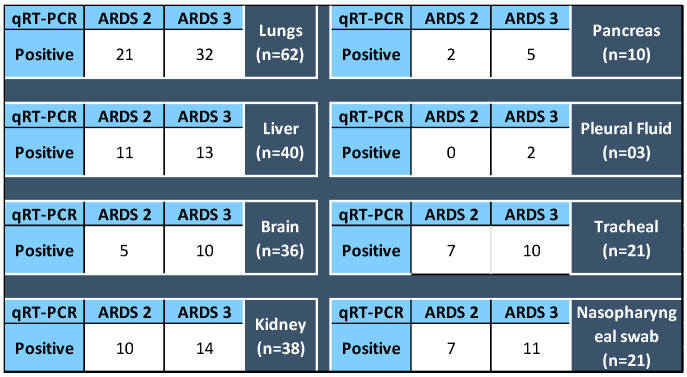
For each organ (total of samples tested for qRT-PCR), the number of positive detected in specimens collected from the autopsy of ARDS 2 and 3 category patients. *p* value for Fisher’s Exact Test > 0.05.

The SARS-CoV-2 detection in various organs was analyzed for their association, if any, with the ARDS category of the patients at the time of admission. It was found that 51.6% SARS-CoV-2 positive lung sample qRT-PCR belonged to ARDS 3 category autopsied patients, and likewise 52.38%, nasopharyngeal, 47.61% tracheal, and 32.5% liver samples positive for SARS-CoV-2 respectively belonged to ARDS 3 patients. However, *p* value for Fischer’s exact test was found to be insignificant, concluding that the ARDS categorization of the patients and the organs infected with SARS-CoV-2 are not correlated Figure 8.

## 4. Discussion

The value of clinicopathological autopsy in providing information about pathogenesis/evolution of diseases specially from a relatively novel pathogen is unmatched, and this study was conducted to understand as to how the infection by an airborne pathogen having upper respiratory tract as primary site of infection is resulting in systemic illness as evident from wide array of clinical manifestations. Owing to advances in the diagnostics, now the laboratories have capacities to detect RNA/DNA by molecular techniques and conclusively establish the presence of a pathogen in organs of interest, without carrying out labor-intensive conventional culture methods which lack sensitivity and specificity also in comparison to newer diagnostic methods. Thus, we combined autopsy and qRT-PCR to determine the virulence of SARS-CoV-2 in terms of its ability to disseminate beyond upper respiratory tract. This work was undertaken during the peak of the 1st wave of the COVID-19 pandemic when there was lots of panic due to the lack of information, and even close relatives of the deceased had apprehensions on handling the dead bodies for performing last rites. Advisory bodies such as the Indian Council of Medical Research (ICMR) had advised avoiding autopsies on COVID-19 positive patients and we sought approval from the Institute’s Ethics Committee to perform the autopsies for advancing much-needed scientific knowledge.

Even though, during the study period, the site of study, i.e., All India Institute of Medical Sciences—Bhopal (AIIMS—Bhopal) was declared as a dedicated COVID-19 treatment facility which effectively ruled out patients with other ailments, to ensure enrollment of true COVID-19-positive cases, only those patients were included in the study in whom SARS-CoV-2 was detected from nasal/oropharyngeal swabs by RT-PCR as mandated by the Ministry of Health (Government of India) and ICMR-prescribed testing methods. The containment of infection was ensured by performing autopsies in a facility equipped with HVAC providing 12 air changes per hour, minimizing aerosol generation and ensuring compliance to prescribed PPE. Uniformity was ensured in sample collection method, quantity of sample, its dilution in preservative transport medium, storage conditions to minimize variables having effect on Ct value.

The central question which we tried to answer through this study was that whether SARS-CoV-2 spreads beyond nasopharynx, because patients have been presenting with varied symptoms suggestive of multi-organ involvement and overwhelming cytokine storm indicative of systemic immune response also. Further, we had scientific apprehension about the possibility of generalized/organ-specific long COVID illness among survivors and this could also be addressed only if the presence of SARS-CoV-2 could be established in vital organs in addition to the upper respiratory tract.

SARS-CoV-2 was conclusively detected by qRT-PCR in our study in lungs, kidneys, liver, spleen, pancreas, brain, peritoneal fluid, plural fluid, pericardial fluid, and even in uterus. The current knowledge states that SARS-CoV-2 interacts with an angiotensin-converting enzyme 2 (ACE2); a protease such as TMPRSS2 or cathepsin elicits cytokines locally to trigger a cascade, resulting in a systemic cytokine storm. While the pathogenesis and treatment modalities are largely focused on respiratory illness, patients are increasingly presenting with symptoms suggesting involvement of neurological, renal, hepatobiliary, cardiac, and gastrointestinal systems. Even though pathophysiological mechanism including mode of spread is poorly understood, viremia, viral-induced thrombosis, and edema have been proposed as possibilities along with subsequent viral multiplication in various organs because ACE-2 receptors have been detected in multiple organs with highest expression seen in oral cavity and gastrointestinal tract. Replication at multiple organ sites will also enhance probability of transmission to new hosts. In the scientific literature published till now, qRT-PCR and microscopy (electron and fluorescence) have been the most common techniques for SARS-CoV detection and both these techniques, though they conclusively establish the presence of the virus, cannot provide evidence of infectious virus.

SARS-CoV-2 was detected in our study from nasopharynx, trachea, and bilateral lung tissues, suggesting the involvement of the upper as well as lower respiratory tract, which in all probability is the reason for the predominance of respiratory symptoms and ARDS. It has been reported in meta-analysis that approximately 20% of COVID-19 patients in ICU developed acute cardiac injury and in our study SARS-CoV-2 was detected in one patient’s pericardial fluid sample [38]. The postmortem study conclusively establishes the presence of the virus in the heart, and the mechanism of injury requires further exploration to resolve whether the cardiac injury is an outcome of direct infection by SARS-CoV-2 or an outcome of inflammation due to disproportionate immune activation. SARS-CoV-2 was detected in liver, pancreas, spleen, and peritoneal fluid in our study and this is probably reflection of the high expression of ACE-2 receptors in epithelial cells in the entire gastrointestinal tract. Liver injury with deranged liver enzymes have been found in 35–56% of COVID-19 patients and, in another study, pancreatic injury (as indicated by elevated amylase or lipase enzymes) is reported in 10% of COVID-19 patients [39,40].

Though mitochondrial swelling, endoplasmic reticulum dilatation, glycogen granule depletion in hepatocytes [41], and raised alanine and aspartate aminotransferases have been found in COVID-19 patients in many studies, suggesting [42,43] that liver infection contributes to hepatic impairment in COVID-19 patients, there was no robust data on presence of SARS-CoV-2 in solid organs, e.g., liver and pancreas, despite the presence of ACE-2 in these organs. We hereby report spatial evidence of SARS-CoV-2 by qRT-PCR in liver tissue in 81% of cases.

Presence of proteins, leucocytes, and RBCs have been found in the urine of up to 75% of COVID-19 patients [44,45] and approximately 25% of hospitalized COVID-19 patients develop acute renal failure, indicating kidney being a major target organ, again presumably due to highly expressed ACE-2 in proximal tubular cells. It has been proposed that kidneys may be a long-term source of SARS-CoV-2 because of viral shedding in urine after cessation of respiratory shedding [46], but it was doubted because the viral RNA was detected in the urine of only 3–4% of COVID-19 patients [47,48,49]. We tend to concur with the notion that SARS-CoV-2 exhibits tropism for kidneys, because in our study it was detected in approximately 15% of kidney samples. Similarly, a very significant number of COVID-19 patients (up to 78%) have symptoms of neurological involvement, ranging from headaches, anosmia, and ageusia to impaired consciousness and convulsions [50,51], which could be ascribed to pathology in the brain and at spinal level. It is yet to be explored whether nervous system pathology is due to direct damage inflicted by the virus, because in our study we could detect SARS-CoV-2 from approximately 46% of brain tissue samples, or due to indirect injury resulting from inflammatory cyto/chemokines. Though conclusive evidence on the mechanism is lacking, it is prudent to note that ACE-2 is expressed in spinal cord and brainstem also, and it is definitively evident from our study that SARS-CoV-2 has the ability to cross blood–brain barrier to reach brain tissue. We would like to emphasize here that while performing autopsy it was ensured that there was no carryover of body fluids from one organ to another, hence detecting the presence of SARS-CoV-2 in various organs is a true reflection of its spatial presence arising due to its invading capacity from the primary site of infection, i.e., the upper respiratory tract.

It is evident that pathogenesis of COVID-19 is not restricted to the respiratory system, but it is an illness affecting multiple body organs, and systemic inflammatory response is also due to multi-organ involvement. Hence, the treatment strategies should also be able to address the involvement of various organ systems. We infer from our study that higher viral load in lungs is perhaps directly proportional to the probability of spread to other vital organs, and patients with higher viral load in respiratory samples (low Ct value) should be more aggressively treated/monitored for any warning signs indicating deteriorating clinical condition. Evidence of the spatial presence of SARS-CoV-2 in various extrapulmonary organs will help in making therapeutic strategies for managing acute SARS-CoV-2 infected patients and also a long-term follow-up framework and case management in COVID-19 patients.

qRT-PCR is one of the important assays to diagnose COVID-19 infection by detecting SARS-CoV-2 viral RNA in given specimen [52,53], and this technique is successful in identifying microorganisms that are difficult to culture in vitro or in situations wherein the culture procedure lacks sensitivity and/or requires sophisticated laboratory setup and prolonged incubation periods [54]. The viral load can be determined to a reasonable extent by the Ct value, which is inversely proportional to the viral load in the sample. Bullard J et al. (2020) determined that, with every unit rise in Ct value, there is 32% rise in the odd ratio of infectivity [55], whereas Singanayagam A et al. estimated the association between Ct value and virus isolation from infected specimen and mentioned that with each unit increase in the Ct value, the recovery of infectious virus decreases by 0.67 [13]. Nevertheless, the Ct value should not be interpreted as an independent unit of pathogen load without a standard curve because the quantification and precision associated with differences in Ct values have not been determined clinically and could not corelate with disease severity. It may be because of variables such as equipment calibration, sample type and quality, scientific competency of test performer, and lastly interpretation skill of the test analyzer considerably affect the Ct value in addition to some inhibitory substances affecting the reaction efficiency significantly and, thus, eventually the final result interpretation.

We acknowledge that while we could demonstrate presence of SARS-CoV-2 in multiple vital organs in addition to the upper respiratory tract and lungs, the transmissibility of virus particles could not be established in corpses due to limitations inherent to the technique of qRT-PCR, and the question of how long SARS-CoV-2 remains viable after death remains unanswered. Hence, it is important to have followed dead-body-management protocols, including the wiping of externally exposed surfaces of the dead body with hypochlorite/other suitable disinfectant, wearing PPE by body handlers, and shifting the body in disinfectant-coated body bags.

Further, there is no precedence available in the published scientific literature for classifying patients to high-, intermediate-, and low-intensity cases based on the number of organs involved, and we are of the opinion that this can serve as a reasonable analysis tool in future autopsy studies.

## 5. Conclusions

SARS-CoV-2 has the ability to disseminate from the upper respiratory tract, which is the primary site of infection to various organs, signifying its virulence in terms of invading property and the systemic illness and profound inflammatory response is an outcome of multi-organ involvement. There had been strong suspicion about kidneys, liver, and brain being the target organs due to the presenting symptoms and laboratory markers, and this has been confirmed in our study due to the detection of SARS-CoV-2 from samples obtained from these organs of those succumbing to severe COVID-19 illness, though it is yet to be established whether the organ damage is due to direct damage caused by the virus or result of inflammatory assault. Irrespective of the mechanism, the treating physicians need to adopt treatment strategies not solely focused on lungs and, as evident from observations in our study, patients with higher viral load in respiratory samples (low Ct value) are more likely to have dissemination to other organs, and should be more closely monitored during phase of acute illness and the probability of organ damage due to SARS-CoV-2 should be kept in mind, should these patients present with organ failure in future.

## Data Availability

Not applicable.

## Supplementary Materials

The following supporting information can be downloaded at: https://www.mdpi.com/article/10.3390/microorganisms10071333/s1, Table S1: The Clinical Details of 21 cases along the the molecular diagnosis (qRT-PCR) results for all the 246 organs included in the study; File S1: COVID Autopsy Microbiological study.
