# Supplementary material for: Organ Involvement in COVID-19: A Molecular Investigation of Autopsied Patients
Source: Microorganisms. 2022 Jul 1;10(7):1333. doi: 10.3390/microorganisms10071333 (PMC9318581; doi:10.3390/microorganisms10071333)
Supplement: Supplementary file 1 [file microorganisms-10-01333-s001.zip › microorganisms-1717321-supplementary.pdf]

Supplementary Table S1. The Clinical Details of 21 cases along the the molecular diagnosis (qRT-PCR) results for all the 246 organs sincluded in the study.

S. No	Lab-ID	Date	Name	Age	Gender	Hospitaliz ation Days	ARDS*	Unique Organ ID	Organ/Body Fluids	Gene of Interest		Qualititati ve Inference
										ORF (Confirmatory)	RNP (For QC)	
1	[redacted]	16.08.2020	CASE-01	58	M	16	2	D-41	Kidney	28.08	17.59	Positive
2	[redacted]							D-42	Liver	28.56	17.22	Positive
3	[redacted]							D-43	Brain	31.96	18.06	Positive
4	[redacted]							D-44	Trachea	31.32	18.13	Positive
5	[redacted]							D-45	Lung	28.20	15.87	Positive
6	[redacted]							D-46	Nasopharynx	26.55	23.10	Positive
7	[redacted]	26.08.2020	CASE-02	67	M	6	2	B-14	Kidney	NA	38.10	Negative
8	[redacted]							B-15	Kidney	35.15	7.34	Positive
9	[redacted]							B-16	Brain	NA	12.60	Negative
10	[redacted]							B-18	Liver	25.59	16.80	Positive
11	[redacted]							B-19	Liver	37.70	16.72	Positive
12	[redacted]							B-20	Trachea	33.28	21.36	Positive
13	[redacted]							B-21	Lung	22.34	13.77	Positive
14	[redacted]							B-22	Lung	28.92	19.85	Positive
15	[redacted]							B-23	Lung	29.45	17.84	Positive
16	[redacted]							B-24	Lung	34.60	29.13	Positive
17	[redacted]							B-25	Nasopharynx	26.01	18.58	Positive
18	[redacted]	26.08.2020	CASE-03	25	M	14	3	A-2	Kidney	29.30	20.74	Positive
19	[redacted]							A-3	Brain	NA	15.88	Negative
20	[redacted]							A-4	Brain	NA	11.90	Negative
21	[redacted]							A-6	Liver	NA	18.63	Negative
22	[redacted]							A-7	Lung	20.62	13.73	Positive
23	[redacted]							A-8	Lung	21.58	14.04	Positive
24	[redacted]							A-9	Lung	29.38	21.28	Positive
25	[redacted]							A-10	Trachea	28.99	16.29	Positive
26	[redacted]							A-11	Nasopharynx	27.24	19.11	Positive
27	[redacted]							A-12	Peritoneal Fluid	32.24	12.68	Positive
28	[redacted]							A-13	Peritoneal Fluid	30.74	19.03	Positive
29	[redacted]	28.08.2020	CASE-04	30	M	2	3	C-26	Brain	33.01	25.84	Positive
30	[redacted]							C-27	Brain	22.92	15.06	Positive
31	[redacted]							C-28	Liver	14.65	16.10	Positive

S. No	Lab-ID	Date	Name	Age	Gender	Hospitalization Days	ARDS*	Unique Organ ID	Organ/Body Fluids	Gene of Interest		Qualititative Inference
										ORF (Confirmatory)	RNP (For QC)	
32	[redacted]							C-29	Liver	24.30	26.33	Positive
33	[redacted]							C-30	Spleen	19.76	17.10	Positive
34	[redacted]							C-31	Lung	19.74	27.00	Positive
35	[redacted]							C-32	Lung	20.48	27.83	Positive
36	[redacted]							C-33	Kidney	14.56	10.73	Positive
37	[redacted]							C-34	Kidney	26.39	27.28	Positive
38	[redacted]							C-35	Lung	23.15	8.80	Positive
39	[redacted]							C-36	Nasopharynx	20.84	18.52	Positive
40	[redacted]							C-37	Trachea	14.25	17.36	Positive
41	[redacted]							C-38	Pleural Fluid	25.35	18.88	Positive
42	[redacted]							C-39	Peritoneal Fluid	28.40	18.76	Positive
43	[redacted]	01.09.2020	CASE-05	60	M	39	3	G-54	Peritoneal Fluid	29.99	20.39	Positive
44	[redacted]							O-63	Liver	NA	24.45	Negative
45	[redacted]							O-64	Lung	25.02	11.87	Positive
46	[redacted]							O-65	Nasopharynx	36.93	21.88	Positive
47	[redacted]							O-66	Lung	NA	24.19	Negative
48	[redacted]							O-67	Liver	NA	25.07	Negative
49	[redacted]							O-68	Lung	36.18	24.26	Positive
50	[redacted]							O-69	Trachea	NA	28.53	Negative
51	[redacted]							O-71	Brain	28.50	17.67	Positive
52	[redacted]							O-72	Kidney	NA	15.08	Negative
53	[redacted]							O-73	Kidney	NA	25.53	Negative
54	[redacted]	02.09.2020	CASE-06	30	M	4	2	K-59	Peritoneal Fluid	30.95	21.36	Positive
55	[redacted]							Y-186	Lung	19.96	22.20	Positive
56	[redacted]							Y-187	Brain	NA	22.16	Negative
57	[redacted]							Y-188	Liver	30.13	25.87	Positive
58	[redacted]							Y-189	Lung	23.02	18.25	Positive
59	[redacted]							Y-190	Brain	37.96	26.41	Positive
60	[redacted]							Y-191	Lung	23.56	23.81	Positive
61	[redacted]							Y-192	Nasopharynx	19.19	22.79	Positive
62	[redacted]							Y-193	Kidney	26.51	24.66	Positive
63	[redacted]							Y-194	Liver	NA	16.18	Negative
64	[redacted]							Y-195	Trachea	21.85	24.45	Positive

S. No	Lab-ID	Date	Name	Age	Gender	Hospitalization Days	ARDS*	Unique Organ ID	Organ/Body Fluids	Gene of Interest		Qualititative Inference
										ORF (Confirmatory)	RNP (For QC)	
65	[redacted]							Y-196	Kidney	16.16	18.95	Positive
66	[redacted]	31.09.2020	CASE-07	51	M	3	3	J-58	Peritoneal Fluid	NA	24.09	Negative
67	[redacted]							T-125	Kidney	25.19	15.14	Positive
68	[redacted]							T-126	Liver	28.78	21.02	Positive
69	[redacted]							T-127	Brain	NA	27.16	Negative
70	[redacted]							T-128	Kidney	30.90	24.84	Positive
71	[redacted]							T-129	Lung	20.80	84.80	Positive
72	[redacted]							T-130	Lung	18.96	23.51	Positive
73	[redacted]							T-131	Lung	18.27	19.34	Positive
74	[redacted]							T-132	Brain	26.72	20.43	Positive
75	[redacted]							T-133	Nasopharynx	27.23	25.91	Positive
76	[redacted]							T-134	Liver	31.48	24.76	Positive
77	[redacted]							T-135	Trachea	28.43	25.55	Positive
78	[redacted]	04.09.2020	CASE-08	75	M	6	2	b-223	Lung	30.71	19.38	Positive
79	[redacted]							b-224	Trachea	NA	14.41	Negative
80	[redacted]							b-225	Lung	NA	8.20	Negative
81	[redacted]							b-226	Pericardial Fluid	35.25	22.27	Positive
82	[redacted]							b-227	Kidney	NA	20.87	Negative
83	[redacted]							b-229	Brain	32.02	9.19	Positive
84	[redacted]							b-230	Liver	27.33	18.36	Positive
85	[redacted]							b-231	Nasopharynx	25.73	17.33	Positive
86	[redacted]							b-232	Lung	NA	23.71	Negative
87	[redacted]							b-233	Liver	31.02	22.79	Positive
88	[redacted]							b-234	Kidney	19.56	13.64	Positive
89	[redacted]	07.09.2020	CASE-09	64	M	9	2	Z-198	Liver	36.61	20.99	Positive
90	[redacted]							Z-199	Liver	NA	33.99	Negative
91	[redacted]							Z-200	Lung	33.86	33.96	Positive
92	[redacted]							Z-201	Nasopharynx	NA	23.75	Negative
93	[redacted]							Z-202	Brain	NA	27.81	Negative
94	[redacted]							Z-203	Pericardial Fluid	NA	32.26	Negative
95	[redacted]							Z-204	Lung	34.09	24.15	Positive
96	[redacted]							Z-206	Trachea	NA	24.93	Negative
97	[redacted]							Z-207	Kidney	NA	23.76	Negative

S. No	Lab-ID	Date	Name	Age	Gender	Hospitalization Days	ARDS*	Unique Organ ID	Organ/Body Fluids	Gene of Interest		Qualititative Inference
										ORF (Confirmatory)	RNP (For QC)	
98	[redacted]							Z-208	Lung	38.05	22.17	Positive
99	[redacted]	08.09.2020	CASE-10	71	M	0	NA	U-137	Lung	10.14	9.48	Positive
100	[redacted]							U-138	Nasopharynx	22.93	26.25	Positive
101	[redacted]							U-139	Kidney	25.87	20.28	Positive
102	[redacted]							U-140	Pericardial Fluid	23.60	19.56	Positive
103	[redacted]							U-141	Lung	18.04	25.88	Positive
104	[redacted]							U-142	Kidney	29.55	26.00	Positive
105	[redacted]							U-143	Liver	21.70	20.81	Positive
106	[redacted]							U-144	Brain	25.77	21.76	Positive
107	[redacted]							U-145	Liver	27.40	25.48	Positive
108	[redacted]							U-146	Trachea	20.53	27.69	Positive
109	[redacted]							U-147	Brain	36.19	26.59	Positive
110	[redacted]	09.09.2020	CASE-11	62	M	5	3	H-55	Peritoneal Fluid	NA	20.79	Negative
111	[redacted]							P-74	Pericardial Fluid	23.67	22.69	Positive
112	[redacted]							P-75	Kidney	36.24	23.39	Positive
113	[redacted]							P-76	Brain	NA	24.87	Negative
114	[redacted]							P-77	Lung	27.65	24.82	Positive
115	[redacted]							P-78	Lung	17.95	13.79	Positive
116	[redacted]							P-79	Nasopharynx	22.40	22.97	Positive
117	[redacted]							P-80	Kidney	22.83	19.44	Positive
118	[redacted]							P-81	Brain	NA	20.74	Negative
119	[redacted]							P-82	Lung	23.18	23.99	Positive
120	[redacted]							P-83	Trachea	31.29	22.74	Positive
121	[redacted]							P-84	Liver	30.18	17.81	Positive
122	[redacted]							P-85	Liver	NA	28.16	Negative
123	[redacted]	16.09.2020	CASE-12	79	M	8	3	W-160	Nasopharynx	35.65	23.21	Positive
124	[redacted]							W-161	Brain	38.80	26.11	Positive
125	[redacted]							W-162	Kidney	NA	24.89	Negative
126	[redacted]							W-163	Lung	21.37	16.74	Positive
127	[redacted]							W-164	Liver	38.77	24.21	Positive
128	[redacted]							W-165	Liver	29.12	18.99	Positive
129	[redacted]							W-166	Brain	NA	17.48	Negative
130	[redacted]							W-167	Pericardial Fluid	32.83	20.67	Positive

S. No	Lab-ID	Date	Name	Age	Gender	Hospitalization Days	ARDS*	Unique Organ ID	Organ/Body Fluids	Gene of Interest		Qualititative Inference
										ORF (Confirmatory)	RNP (For QC)	
131	[redacted]							W-168	Lung	31.50	23.67	Positive
132	[redacted]							W-169	Trachea	34.66	23.19	Positive
133	[redacted]							W-170	Kidney	34.71	19.52	Positive
134	[redacted]							W-171	Lung	31.70	23.37	Positive
135	[redacted]	17.09.2020	CASE-13	45	F	2	3	X-172	Uterus	30.42	15.42	Positive
136	[redacted]							X-173	Liver	33.34	24.45	Positive
137	[redacted]							X-174	Kidney	33.11	23.42	Positive
138	[redacted]							X-175	Brain	NA	24.69	Negative
139	[redacted]							X-176	Liver	34.18	23.17	Positive
140	[redacted]							X-177	Pericardial Fluid	28.23	23.98	Positive
141	[redacted]							X-178	Kidney	30.52	18.86	Positive
142	[redacted]							X-179	Lung	26.10	21.66	Positive
143	[redacted]							X-180	Brain	NA	19.44	Negative
144	[redacted]							X-181	Lung	NA	23.69	Negative
145	[redacted]							X-182	Nasopharynx	26.33	23.24	Positive
146	[redacted]							X-183	Trachea	29.93	21.76	Positive
147	[redacted]							X-184	Lung	25.48	20.81	Positive
148	[redacted]							E-48	Peritoneal Fluid	22.34	22.45	Positive
149	[redacted]							E-49	Pericardial Fluid	30.86	18.50	Positive
150	[redacted]	22.09.2020	CASE-14	75	M	8	2	F-52	Peritoneal Fluid	29.73	26.10	Positive
151	[redacted]							V-148	Trachea	36.23	24.91	Positive
152	[redacted]							V-150	Brain	NA	25.42	Negative
153	[redacted]							V-151	Liver	NA	22.44	Negative
154	[redacted]							V-152	Liver	25.68	21.48	Positive
155	[redacted]							V-153	Lung	30.39	23.84	Positive
156	[redacted]							V-154	Kidney	33.91	24.32	Positive
157	[redacted]							V-155	Nasopharynx	33.42	24.79	Positive
158	[redacted]							V-156	Lung	33.00	23.42	Positive
159	[redacted]							V-157	Kidney	24.81	18.83	Positive
160	[redacted]							V-158	Lung	NA	17.15	Negative
161	[redacted]	12.09.2020	CASE-15	70	F	10	3	S-113	Liver	NA	24.09	Negative
162	[redacted]							S-114	Lung	29.33	23.95	Positive
163	[redacted]							S-115	Liver	30.19	18.19	Positive

S. No	Lab-ID	Date	Name	Age	Gender	Hospitaliz ation Days	ARDS*	Unique Organ ID	Organ/Body Fluids	Gene of Interest		Qualititati ve Inference
										ORF (Confirmatory)	RNP (For QC)	
164	[redacted]							S-116	Kidney	26.77	17.79	Positive
165	[redacted]							S-117	Pericardial Fluid	26.52	24.45	Positive
166	[redacted]							S-118	Lung	26.15	23.97	Positive
167	[redacted]							S-119	Kidney	NA	25.43	Negative
168	[redacted]							S-120	Brain	32.12	22.77	Positive
169	[redacted]							S-121	Brain	39.75	25.85	Positive
170	[redacted]							S-122	Nasopharynx	25.85	24.34	Positive
171	[redacted]							S-123	Trachea	25.76	25.94	Positive
172	[redacted]							S-124	Lung	19.60	7.92	Positive
173	[redacted]	04.09.2020	CASE-16	84	M	10	2	c-236	Trachea	19.33	20.29	Positive
174	[redacted]							c-237	Pericardial Fluid	NA	26.16	Negative
175	[redacted]							c-238	Nasopharynx	29.19	19.69	Positive
176	[redacted]							c-239	Liver	NA	22.20	Negative
177	[redacted]							c-240	Lung	22.65	19.58	Positive
178	[redacted]							c-241	Liver	29.02	18.66	Positive
179	[redacted]							c-242	Kidney	NA	15.19	Negative
180	[redacted]							c-244	Lung	21.27	10.12	Positive
181	[redacted]							c-245	Brain	NA	20.86	Negative
182	[redacted]	04.09.2020	CASE-17	75	F	9	2	R-101	Liver	NA	25.86	Negative
183	[redacted]							R-102	Pericardial Fluid	24.05	24.00	Positive
184	[redacted]							R-103	Brain	39.01	25.92	Positive
185	[redacted]							R-104	Lung	15.34	12.94	Positive
186	[redacted]							R-105	Kidney	31.97	24.29	Positive
187	[redacted]							R-106	Nasopharynx	21.84	22.62	Positive
188	[redacted]							R-107	Lung	28.14	25.12	Positive
189	[redacted]							R-108	Trachea	26.73	24.60	Positive
190	[redacted]							R-109	Brain	NA	34.50	Negative
191	[redacted]							R-110	Lung	26.41	25.27	Positive
192	[redacted]							R-111	Liver	30.10	18.55	Positive
193	[redacted]							Rx	Kidney	30.30	24.67	Positive
194	[redacted]	30.09.2020	CASE-18	72	M	20	3	a-209	Liver	NA	22.15	Negative
195	[redacted]							a-210	Lung	25.75	20.50	Positive
196	[redacted]							a-211	Brain	28.44	12.44	Positive

S. No	Lab-ID	Date	Name	Age	Gender	Hospitalization Days	ARDS*	Unique Organ ID	Organ/Body Fluids	Gene of Interest		Qualititative Inference
										ORF (Confirmatory)	RNP (For QC)	
197	[redacted]							a-212	Kidney	39.65	24.46	Positive
198	[redacted]							a-213	Brain	NA	24.97	Negative
199	[redacted]							a-214	Trachea	30.97	23.54	Positive
200	[redacted]							a-215	Lung	NA	25.75	Negative
201	[redacted]							a-216	Nasopharynx	26.16	21.49	Positive
202	[redacted]							a-217	Lung	26.74	19.74	Positive
203	[redacted]							a-219	Liver	NA	13.09	Negative
204	[redacted]							a-220	Lung	21.99	19.46	Positive
205	[redacted]							a-221	Kidney	NA	10.88	Negative
206	[redacted]							a-222	Pleural Fluid	NA	17.15	Negative
207	[redacted]	02.10.2020	CASE-19	64	F	18	3	Q-86	Lung	37.99	24.90	Positive
208	[redacted]							Q-87	Kidney	NA	25.87	Negative
209	[redacted]							Q-88	Lung	36.31	23.34	Positive
210	[redacted]							Q-89	Liver	31.35	18.44	Positive
211	[redacted]							Q-90	Lung	NA	24.34	Negative
212	[redacted]							Q-91	Lung	33.02	15.87	Positive
213	[redacted]							Q-92	Lung	26.92	15.96	Positive
214	[redacted]							Q-93	Brain	24.10	16.52	Positive
215	[redacted]							Q-94	Trachea	36.67	23.92	Positive
216	[redacted]							Q-95	Nasopharynx	32.90	22.16	Positive
217	[redacted]							Q-96	Liver	38.29	23.29	Positive
218	[redacted]							Q-97	Kidney	NA	16.45	Negative
219	[redacted]							Q-98	Brain	NA	23.96	Negative
220	[redacted]							Q-99	Pericardial Fluid	NA	28.56	Negative
221	[redacted]							Q-100	Pleural Fluid	33.66	22.22	Positive
222	[redacted]	21.10.2020	CASE-20	60	F	3	2	β-1	Kidney	31.80	29.83	Positive
223	[redacted]							β-2	Nasopharynx	NA	28.00	Negative
224	[redacted]							β-3	Lung	28.94	28.03	Positive
225	[redacted]							β-4	Lung	NA	32.71	Negative
226	[redacted]							β-5	Brain	33.04	32.04	Positive
227	[redacted]							β-6	Brain	NA	27.91	Negative
228	[redacted]							β-7	Liver	NA	35.40	Negative
229	[redacted]							β-8	Liver	32.25	26.85	Positive

S. No	Lab-ID	Date	Name	Age	Gender	Hospitalization Days	ARDS*	Unique Organ ID	Organ/Body Fluids	Gene of Interest		Qualititative Inference
										ORF (Confirmatory)	RNP (For QC)	
230	[redacted]							β-9	Pericardial Fluid	NA	34.04	Negative
231	[redacted]							β-10	Lung	28.62	25.31	Positive
232	[redacted]							β-11	Trachea	28.46	26.73	Positive
233	[redacted]	28.10.2020	CASE-21	60	F	4	3	α-1	Liver	34.33	27.24	Positive
234	[redacted]							α-2	Brain	NA	27.05	Negative
235	[redacted]							α-3	Liver	NA	25.35	Negative
236	[redacted]							α-4	Trachea	32.55	26.04	Positive
237	[redacted]							α-5	Kidney	NA	27.30	Negative
238	[redacted]							α-6	Lung	28.20	26.03	Positive
239	[redacted]							α-7	Lung	27.73	27.42	Positive
240	[redacted]							α-8	Brain	32.40	27.03	Positive
241	[redacted]							α-9	Nasopharynx	31.18	25.82	Positive
242	[redacted]							α-10	Pericardial Fluid	32.81	35.53	Positive
243	[redacted]							α-11	Lung	34.80	24.82	Positive
244	[redacted]							α-12	Kidney	29.47	26.46	Positive
245	[redacted]							γ-2	Kidney	33.92	26.45	Positive
246	[redacted]							γ-4	Brain	NA	37.54	Negative

*ARDS Categorization of the cases was done at the time of admission to the hospital, Case-10 was declared brought dead, therefore not categorized.

ARDS- Acute Respiratory Distress Syndrome

Supplementary File S1. COVID Autopsy Microbiological study.

```
---

title: "COVID Autopsy Microbiological study"

output:

  word_document: default

  pdf_document: default

---


```{r setup, include=FALSE}


knitr::opts_chunk$set(eval = FALSE)



library(dplyr)

library(ggplot2)

library(readxl)

library(gtsummary)

library(jpeg)

library(png)

library(knitr)

library(ggpubr)

library(rstatix)

library(dplyr)

library(ggplot2)
```
```

```
library(readxl)

library(gtsummary)

library(jpeg)

library(png)

library(knitr)

library(ggpubr)

library(rstatix)

df1 <- read_excel("~/Desktop/Autopsy.xlsx")

df1<-df1[complete.cases(df1),]

```

data cleaning and changing the variable

```{r mplt}

summary(df1)

x<-c("uid","organ_id" , "result")

df1[,x]<-lapply(df1[,x],factor)

str(df1)

df1%>% select(uid,Organ,result )%>%group_by(uid,result)%>%tbl_summary(by=result,percent = "row")

df1%>%filter(result=="Positive")%>%

  select(Organ,result,RNP_value )%>%

  group_by(Organ)%>%
```

````
  dplyr::summarize(Count=n(),mean_ct = mean(RNP_value, na.rm = TRUE),std_ct=sd(RNP_value, na.rm = TRUE),median_ct=median(RNP_value, na.rm = TRUE),iqr_ct=IQR(RNP_value, na.rm = TRUE))

##create a percent site coloumn

df1 %>% select(1,3,4,5) ->df2

df2 %>%

  group_by( uid ) %>%

  summarise(Count=n(), Percent_site = length( result[ which( result == "Positive") ] )/n()) %>%

  arrange( desc( Percent_site ) )->d

data <- left_join(df1,d, by="uid")

  data %>% mutate(Intensity=case_when(Percent_site>0.80~"High_intensity",

                  Percent_site<0.80 & Percent_site<0.60~ "Intermediated_intensity" ,TRUE~"Low_intensity" ))->data

   data$Intensity<-ordered(data$Intensity, levels = c("Low_intensity","Intermediated_intensity","High_intensity"),labels=c("Low_intensity","Intermediated_intensity","High_intensity"))


```



## PLot



```{r plot, echo=FALSE,fig.width=12,fig.height=6,dpi = 200,out.width='70%'}

data %>% select(3,4,5,8)->data1

data1 %>% group_by(Organ,result,Intensity) %>% summarise(Count=n())->data2

# Sort by the Organ and result columns

data2 <- data2 %>%

  arrange(Organ,result)

# Get the cumulative sum
````

~~~~
library(ggthemes)

data2<- data2 %>%

  group_by(Organ) %>%

  mutate(label_y = cumsum(Count))

data2%>% ggplot(aes(x =Organ,y=Count ,fill=result))+geom_col(position = "dodge")+facet_wrap(~Intensity, scales = "free_x")+

  geom_text(

    aes(label = Count),

    colour = "black", size = 2,face="bold",

    vjust = 1

  )+

  coord_flip()+theme_cleveland()









###BOXPLOT

  df1$percent_sites<-ordered(df1$percent_sites, levels = c("40-60%", "60-80%", ">80%"),labels=c("low intensity","intermediated intensity","high intensity"))

df1%>%filter(result=="Positive")%>% select(organ,ct_value,percent_sites )%>%ggplot(aes(x=percent_sites,y=ct_value,fill=organ))+geom_boxplot()+scale_color_brewer(palette="Dark2")+labs(x="Intensity infection",y="CT values")+ggtitle("Boxplot-CTvalues of infection organs as per intensity of infection")+

  theme_classic()

```
~~~~

Note that the `echo = FALSE` parameter was added to the code chunk to prevent printing of the R code that generated the plot

````
# After adjusting the effects of organs involved whether intensity of infection is affecting the CT values?

```{r disease}

 df1[complete.cases(df1[,9]), ]->df3

df3 %>% select(uid_io,organ,result,percent_sites,ARDS, Hos_Days) %>% tbl_cross(percent_sites,ARDS,missing="no", percent = "cell") %>% add_p()

df3 %>% filter(result=="Positive"|organ=="Blood") %>% select(uid_io,organ,result,percent_sites,ARDS, Hos_Days) %>% tbl_cross(ARDS,organ,missing="no", percent = "column") %>% add_p()

```
````
